# Dental Epochs in the Nineteenth Century

**Published:** 1900-01

**Authors:** 


### Editorial.

### DENTAL EPOCHS IN THE NINETEENTH CENTURY.

The opening of the last year of the century very naturally and properly leads the mind to the consideration of the influence exerted upon dentistry by the introduction of various things, perhaps small intrinsically, but leading up to great results. It is possible after the lapse of years to judge the effect of certain proceedures, and hence at the close of one hundred years of dental work it is proper to intelligently consider the results in relation to progress in our calling.

It is not difficult to mark certain epochs that have led up to great changes in practice and have made dentistry what it is to-day in America and perhaps in Europe.

When Spooner, of Montreal, Canada, in 1836, gave to dentistry arsenous oxide as a means for the devitalization of the pulp, he could not possibly have conceived of the far-reaching effect that this simple announcement would produce in dental practice. His contemporaries in the profession regarded its introduction as a positive injury, and spurned it to the extent that it required several years to overcome the prejudice of certain leaders in dentistry, and yet the effect of its introduction was not only far reaching, but the good it accomplished has probably no parallel in the use of any other therapeutic agent. Sixty-four years have passed since then, but to-day we are still using this agent, and have not found anything that will take its place with the same degree of certainty in the devitalization of pulps.

When Hudson filled pulp-canals he probably thought nothing of it beyond the fact that it was effectual in producing certain results, and therefore kept it to himself; but Maynard, with a broader mind, perfected this process and gave it freely to the dental profession. Then it was that the value of the Spooner introduction of arsenic became apparent, one naturally leading to the other, for it was the mistaken idea of Harris, and those who thought with him, that it was the arsenic used that led up to violent inflammations rather than the septic conditions of an open canal.

Thus the introduction of arsenous oxide was the first and most important step in the salvation of teeth through the perfect closing of root-canals, by filling, to septic influences, and this must be considered the first and only epoch worthy of note up to this period in the present century.

When Dr. Harris, a year or two later, plead with the faculty of a medical college to establish dental teaching as a branch of medical instruction, he was contemptuously dismissed, and was forced to establish a separate and distinct school. Thus, in 1839, the Baltimore College of Dental Surgery opened its doors, and in that year began a new profession; and to-day, sixty-one years subsequent to this organization, dental education has reached a degree of perfection which Harris could not have conceived in his wildest imaginings. In fact, he died at a period when the few dental colleges were struggling against three almost hopeless conditions,—the prejudice of the dentists of the country, the contempt of the medical profession, and pecuniary embarrassments. In spite of all these and more, dental education grew, and to-day medical colleges and universities are eager to have dental departments connected with them, and medical men no longer consider it beneath their dignity to take positions as professors in separate dental schools. Truly time has not only confirmed the wisdom of Dr. Harris, but has heaped coals of fire upon the heads of those narrow-minded men who spurned his offer. Even they builded better than they knew, for had his proposition been accepted it is doubtful whether dental education would have reached its present advanced position. The establishment of the first college of dental surgery led not only to an increase of dental schools, but forced the faculties to increased exertions to constantly perfect the quality of work. The idea of colleges as a means for dental training gradually spread to England and the Continent until at the present time schools are springing up in all civilizations. From the organization of colleges have developed the necessity for laws to govern graduation. Thus Dr. Harris's feeble effort has proved far reaching, and becomes, as we scan the century, the most important tant epoch in the receding years.

In 1854, when Dr. Arthur reported that the cohesive property of gold could be made a valuable aid in filling cavities, neither he nor his colleagues could have anticipated the effect that would be produced in the progress of years. It came to the dentists of the time after many years of the use of non-cohesive gold, and it was, necessarily, coldly received, yet its introduction led up to an entire change of instruments, to the use of the mallet, power mallets, electrical and mechanical, rubber dam, clamps, and a long list of accessories in filling teeth, and finally to the surgical engine of Bonwill. In fact, it may truthfully be said that modern dentistry dates from the period when Arthur brought into practical use the simple and long-known fact that two sheets of gold-foil, fresh from the beater's hands, would cohere without pressure. This, then, must be placed as the third important epoch in dentistry.

The critical mind might object to this classification, for Wells discovered anæsthesia ten years previously,—1844. This is true, but anæsthesia cannot be regarded as marking an important era in dentistry. It belongs more particularly to the realm of surgery. While dentistry glories in the fact that anæsthesia found its birth in its ranks, and Wells and Morton, both dentists, must go down on the pages of history an honor to our profession, it yet remains true that anæsthesia has not been of positive benefit to dental operators. Extraction of teeth is the last resort of dental surgery, and is a discreditable reminder of the limitations of human effort.

The next and most important era is that which began with the so-called germ theory of disease. The discoveries of Pasteur, Koch, together with the special work of Leber and Rottenstein, Milles and Underwood, Miller, and others, led up to important changes in dental practice. This country can lay no claim to this work, but the results of the study of bacteriology have changed the entire therapeutics of dentistry, in fact it may be said to have changed the curricula of our colleges, leading them nearer to the standard of the higher medical schools. While dentistry has benefited by this discovery, it cannot be said that this is to a greater extent than surgery. Probably Lister had no idea that his suggestions as to antiseptic treatment would lead up to the extraordinary operations of the present time, but his work was therefore none the less valuable. It is the first step that marks progress, all others are comparatively simple and easy of attainment.

Before antisepsis was understood the practice of dental therapeutics was pure empiricism, and is to-day where this is not made a special study. The dentist of the earlier part of the last half of this century had no control of a decomposed pulp. He was thankful if this failed to run rapidly into an abscess, for the knowledge to meet these septic conditions was not at hand. Abscesses were the common inheritance of a race then fast becoming edentulous.

Allusion has been made to the introduction of mallets and various machinery, and these necessarily led to electrical motive force and compressed air. All of these are but the outgrowth of the introduction of cohesive gold, and are therefore not to be regarded as specially influencing the dental mind.

Many things are too recent and too near the close of the century to determine their true value, and among these may be classed cataphoresis. The historian of the future can be better able to give this operation its proper position in practical dentistry. The same may be said of Bonwill's surgical engine. It may mark a decided change in many surgical operations and be only second in importance to anæsthesia, but before that is fully determined, men who essay to work with it must understand the use of tools, and this places its universal adoption a long way in the future.

The analysis of the past and its effect upon dentistry is, it appears to the writer, an important part of dental education. It embraces the value of small things. The crude conception of Spooner made dentistry what it is to-day, and possibly some unknown worker may be evolving ideas that will revolutionize the dentistry of the coming century. Our artistic conception of a filling has been based for a hundred years or more on gold. May not the ideal filling-material of the coming century be an entirely different material, and lead us up to a more perfect salvation of teeth with greater comfort to our patients and to the reduction of the excessive labor which belongs to the dental profession as now understood?

Entering upon the last year of the century, we may look forward with assured confidence to the future. We have built the foundations well in this country and have spent the hundred years in perfecting the operative and prosthetic portions of our work. Now, in the future more must devote themselves to the scientific side and endeavor to emulate our European brethren in those things that truly make a profession.

